# Supplementary material for: A method to estimate population densities and electricity consumption from mobile phone data in developing countries
Source: PLoS One. 2020 Jun 30;15(6):e0235224. doi: 10.1371/journal.pone.0235224 (PMC7326166; doi:10.1371/journal.pone.0235224)
Supplement: S1 Appendix — Alternative method to estimate nightlights intensity from approximate data, validation of the performance of the clustering process and additional figures. (PDF) [file pone.0235224.s004.pdf]

A method to estimate population densities and electricity consumption from mobile phone data in developing countries - Supplementary information

Hadrien Salat[1,2*], Zbigniew Smoreda[2], Markus Schläpfer[1]

1 Future Cities Laboratory, Singapore-ETH Centre, ETH Zürich, Singapore, Singapore
2 Sociology and Economics of Networks and Services department, Orange Labs, Châtillon, France

* hadrien.salat@orange.com

Validation of the performance of the clustering process

Assuming that the full data distribution is known beforehand, we define cluster purity conditions and scroll through the dendrogram by splitting branches when they do not meet the specified conditions. The results for some conditions over all 33 trees are shown in table S1. The conditions are formulated as"the cluster must contain at least x_1% of towers with a value between y_0 and y_1 or at least x_2% of towers with a value between y_1 and y_2, etc." The different thresholds for different bins can prove useful if the size of the bins is not homogeneous. We aim at fulfilling the conditions in as few clusters as possible. Once the process has been operated over all trees, a final prediction is computed for each tower. The expected value inside each cluster the tower belongs to (that is one per tree) weighted by the cluster's purity are averaged. We can obtain high r^2 with hard conditions at the cost of a high number of clusters (column 1 of table S1) or a very low number of clusters per tree with softer conditions at the cost of less precision (column 2 of table S1).

Additional cases of dendrogram sampling

Note that for the yearly curves (case (f) of Fig. S1), only the 979 curves with less than 30 days with 0 activity were considered. This is done to compensate for some towers being activated during the study period.

Table S1. Number of clusters necessary to fulfil the required cluster purity conditions. D, W and Y mean daily, weekly or yearly profiles; T, C and L mean text messages, number of calls and call length; Sd and Cor mean that the standard deviation or correlation method was used; 0, 1, 2, 3, 4 in the Network description indicate the threshold used to subdivide the network (see main text). The thresholds are expressed in percent. Only the upper bounds of the bins are indicated.

	Population density			Electricity
Bins	(500,10k,20k,55k)	(500,10k,20k,55k)	(1k,10k,35k,55k)	(10,30,50,63)
Thresholds	(75,55,55,55)	(70,50,50,50)	(82,65,65,70)	(82,60,60,70)
Curve DTSd	81	39	269	182
Curve DTCor	115	26	321	212
Curve DCSd	110	29	224	136
Curve DCCor	97	83	258	163
Curve DLSd	37	5	279	210
Curve DLCor	111	59	273	208
Curve WTSd	61	40	214	215
Curve WTCor	190	104	351	213
Curve WCSd	101	76	207	104
Curve WCCor	60	35	217	140
Curve WLSd	57	41	276	143
Curve WLCor	112	28	252	193
Curve YTSd	156	88	239	197
Curve YTCor	177	118	314	328
Curve YCSd	129	68	248	158
Curve YCCor	213	155	330	300
Curve YLSd	87	58	280	220
Curve YLCor	102	56	268	237
Curve r^2	0.75	0.66	0.91	0.93
Network T0	75	30	261	167
Network T1	69	22	179	182
Network T2	84	62	160	157
Network T3	62	7	186	153
Network T4	116	9	158	170
Network C0	51	11	196	156
Network C1	37	13	151	202
Network C2	47	21	183	194
Network C3	13	10	211	84
Network C4	44	26	201	169
Network L0	127	66	291	217
Network L1	90	68	208	190
Network L2	72	23	207	193
Network L3	146	63	235	217
Network L4	90	27	214	210
Network r^2	0.67	0.53	0.87	0.88

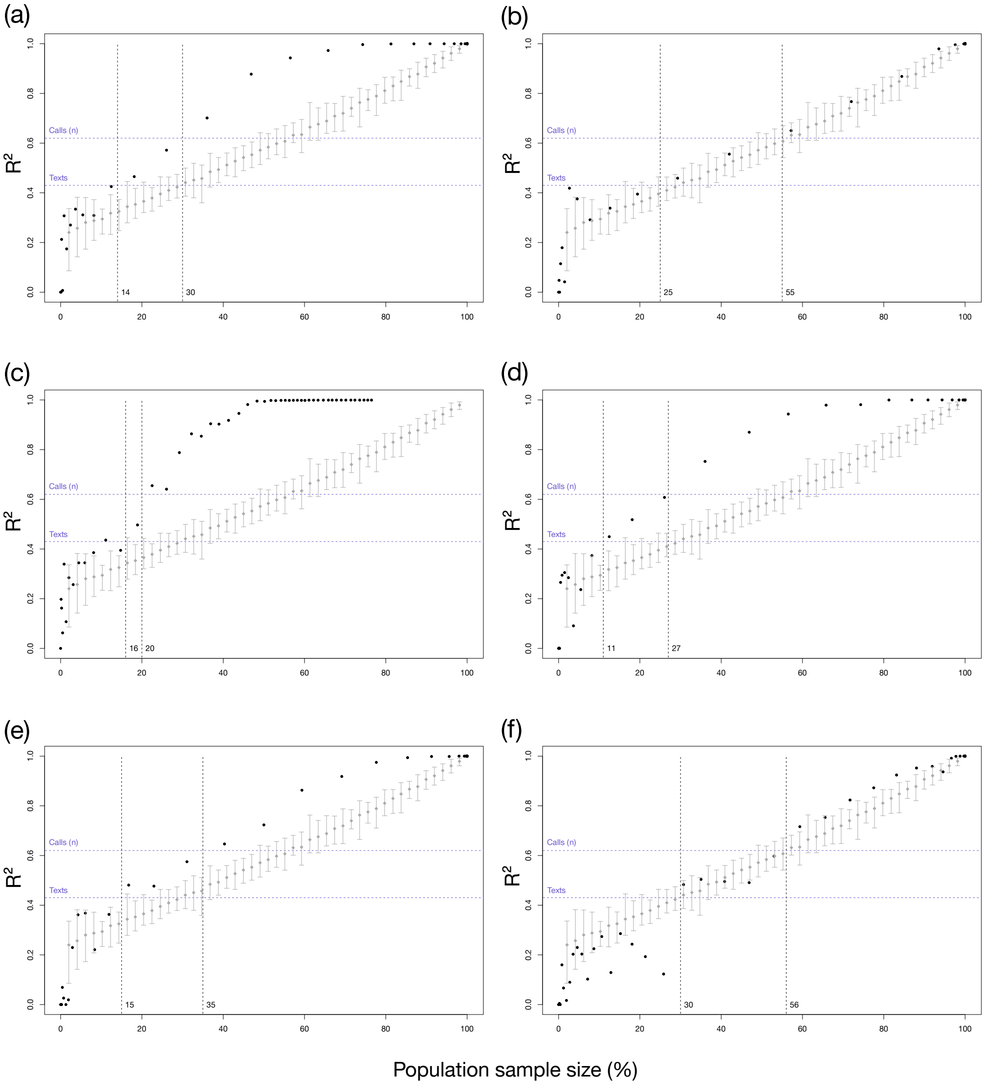

Fig S1. Additional results of density estimations from dendrogram guided sampling. (a) Daily / Length / Sd. (b) Daily / Calls / Cor. (c) Weekly / Texts / Sd. (d) Weekly / Length / Sd. (e) Weekly / Calls / Cor. (f) Yearly/ Length / Sd. The boxes representing random samples have been simplified into error bars for readability.
