# Supplementary material for: A method to estimate population densities and electricity consumption from mobile phone data in developing countries
Source: PLoS One. 2020 Jun 30;15(6):e0235224. doi: 10.1371/journal.pone.0235224 (PMC7326166; doi:10.1371/journal.pone.0235224)
Supplement: Attachment — Submitted filename: Response to Reviewers.pdf [file pone.0235224.s006.pdf]

Hadrien Salat
Orange Labs
44 Avenue de la République
92320 Châtillon, France
hadrien.salat@orange.com

June 8th, 2020

To: *PLoS One* Editorial Office

Dear Dr. Fu,

We thank the Editor and the Reviewers for their new comments and positive feedback.

Editor's comment

In the abstract, "underwhelming" seems inappropriate, what does "a correlation is underwhelming" exactly mean? Please consider rephrasing this.

We have changed "underwhelming" to "insufficiently high to provide an accurate representation of the situation" in the abstract.

Reviewer #2's comments

1. The author adds sentences "The map was created by the authors using R", what information authors would like to convey?

During re-submission, the in-house checks revealed that we needed to either "(1) present written permission from the copyright holder to publish [our] figures specifically under the CC BY 4.0 license, or (2) remove the figures from [our] submission". The quoted sentence was added to indicate that the maps do not, in fact, contain any copyrighted material as we created them ourselves. The justification within the submission system is probably enough, so we have removed the two clumsy sentences from the text at this stage.

2. In the description of Figure 3, the best direct correlation from Table ?? for each case is represented..., which Table?

Well spotted, thank you! This has now been fixed.

Other

We have changed the named contact at Sonatel to whom data inquires should be addressed, also requested during the in-house checks, as we are now aware that someone else within Sonatel is a better fit for the role.

Yours sincerely,
Hadrien Salat
(corresponding author)
